# Abdominal Pain Unveiled: Torsion of a Wandering Spleen in a Background of Biliary Stenting

**DOI:** 10.7759/cureus.84819

**Published:** 2025-05-26

**Authors:** Prashant Gautam, Prabha Om, Farukh Khan, Prerna Dhabhai, Ishan Goyal

**Affiliations:** 1 General Surgery, Sawai Man Singh Medical College, Jaipur, IND

**Keywords:** abdominal mass, extrahepatic portal vein obstruction (ehpvo), splenectomy, splenic ischemia, splenic torsion, surgical acute abdomen, wandering spleen

## Abstract

Wandering spleen (WS) is a rare clinical entity caused by congenital or acquired laxity of the splenic ligaments, resulting in abnormal splenic mobility. This condition often remains undiagnosed due to nonspecific symptoms but may lead to life-threatening complications such as torsion and infarction. We report a case of a 23-year-old male with a history of extrahepatic portal vein obstruction and biliary stenting, who presented with acute abdominal pain. Imaging revealed an enlarged spleen with associated ischemic changes. The spleen had migrated to the right iliac fossa, forming a palpable lower abdominal mass. Intraoperatively, dense adhesions and splenic infarction were observed, necessitating a total splenectomy. This case highlights the diagnostic challenges associated with WS, particularly in patients with complex hepatobiliary histories. Early imaging, especially CT, is crucial for diagnosis. Prompt surgical management tailored to splenic viability, splenopexy for a viable spleen, or splenectomy in cases of infarction is essential to prevent severe outcomes. Clinicians should maintain a high index of suspicion for WS in patients presenting with recurrent, unexplained abdominal symptoms.

## Introduction

Wandering spleen (WS) is a rare condition characterized by abnormal splenic mobility resulting from congenital absence or acquired laxity of the suspensory ligaments, including the gastrosplenic, splenorenal, and splenocolic ligaments [1,2]. This allows the spleen to migrate from its normal position in the left upper quadrant to other locations within the abdomen or pelvis. WS is more commonly observed in two groups: children, due to congenital ligamentous malformations, and women of reproductive age, in whom hormonal changes, particularly involving relaxin and abdominal wall laxity during pregnancy, contribute to ligament weakening [3-5].

The clinical presentation varies widely. Some patients remain asymptomatic, while others present with an acute abdomen due to torsion of the splenic vascular pedicle, potentially resulting in ischemia, infarction, or rupture [6-8]. WS may be misdiagnosed as ovarian torsion, mesenteric volvulus, or other intra-abdominal pathologies, especially in emergencies [4,9,10]. One important and underrecognized association is with extrahepatic portal vein obstruction (EHPVO), a condition that causes chronic splenic congestion and splenomegaly due to non-cirrhotic portal hypertension. In such cases, the enlarged spleen can exert excessive tension on its ligaments, potentially contributing to the secondary development of WS [6].

Imaging plays a critical role in diagnosis. Computed tomography (CT) is the gold standard, capable of identifying the ectopic spleen, vascular compromise, and the characteristic "whirl sign" indicative of torsion [11-13]. Ultrasound, particularly with Doppler, may assist in evaluating spleen position and blood flow, though it is often limited in acute settings due to overlying bowel gas [11]. Timely recognition and surgical intervention are essential to prevent serious complications such as infarction, sepsis, or rupture.

## Case presentation

**Figure 1:**
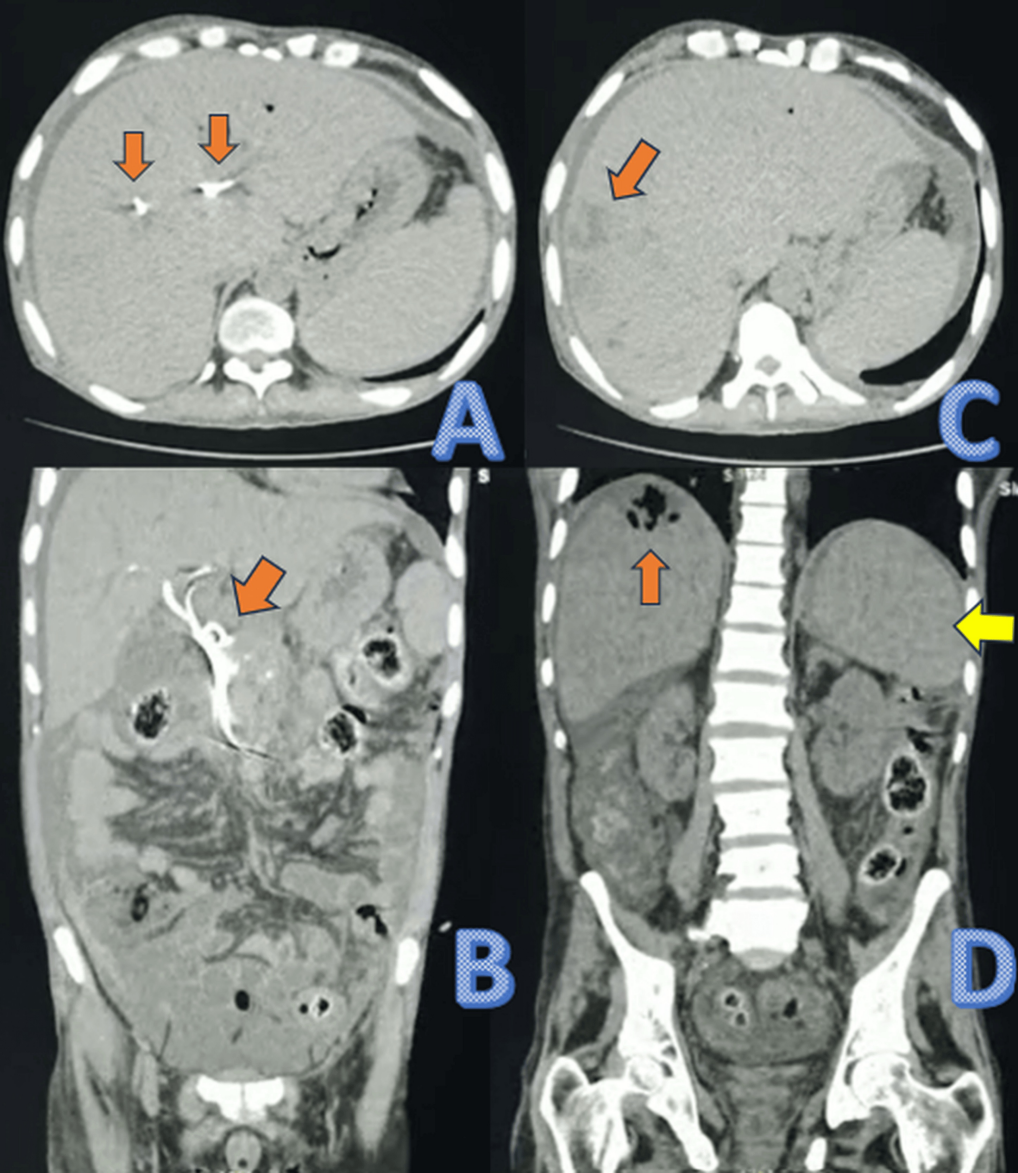
Non-contrast CT scan of the abdomen: (A) Axial CT scan showing biliary stents in situ. (B) Coronal reconstruction highlighting a biliary stent in situ. (C) Axial CT image revealing a hypodense lesion (orange arrow) suggestive of cholangiolar abscesses in the right lobe of the liver. (D) A coronal CT scan showing an orange arrow pointing to cholangiolar abscesses in the right lobe of the liver and a yellow arrow indicating an enlarged spleen CT: computed tomography

A 23-year-old male presented to the emergency department with severe, generalized abdominal pain of one day's duration. He had experienced similar episodes intermittently over the preceding 1.5 years, which were relieved with medications. The current episode was not associated with fever, nausea, vomiting, or constipation. His medical history was notable for EHPVO with associated biliary cholangiopathy, for which he had undergone biliary stenting. On physical examination, a mobile, non-tender, firm lump measuring approximately 12 cm × 9 cm was palpated in the left hypochondrium, extending above the umbilicus, without signs of abdominal guarding or rigidity. Preliminary ultrasonography (USG) performed in the emergency department revealed free fluid in the pelvis and an enlarged spleen measuring approximately 13 cm × 10 cm, with heterogeneous echogenicity suggestive of abnormal splenic parenchyma. Non-contrast CT of the abdomen confirmed a significantly enlarged spleen (14 cm × 12 cm × 7 cm) located in the left hypochondrium and extending below the costal margin. Additionally, multiple cholangiolar abscesses were identified in the right hepatic lobe, likely secondary to stent blockage, with the previously placed biliary stent still in situ (Figure 1).

Laboratory evaluation revealed a hemoglobin concentration of 10.2 g/dL, a total leukocyte count of 4100/mm³, and a platelet count of 54000/mm³, consistent with moderate thrombocytopenia. Liver function parameters were largely within normal limits, with serum bilirubin of 1.1 mg/dL, AST at 38 IU/L, ALT at 34 IU/L, and alkaline phosphatase at 116 IU/L. Pancreatic enzymes, including serum amylase and lipase and other biochemical parameters, were also found to be within the normal range.

**Figure 2:**
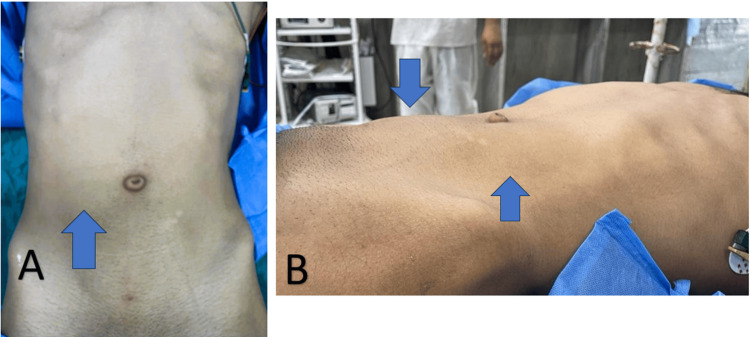
Preoperative clinical presentation shows (A) an anterior view where a visible bulge is noted in the right lower quadrant of the abdomen (arrow), correlating with splenic displacement, and (B) a lateral view where the raised abdominal contour (arrow) supports the suspicion of an ectopically located spleen

The patient subsequently experienced another episode of acute abdominal pain on the next day of admission, now accompanied by a visible lower abdominal lump. On clinical examination, the lump had shifted from the left hypochondrium and extended from above the umbilicus to the right iliac fossa. These findings raised strong suspicion for a WS with torsion of the splenic pedicle (Figure 2).

An exploratory laparotomy was planned (Video 1).

**Video 1:** WS: exploratory laparotomy WS: wandering spleen

**Figure 3:**
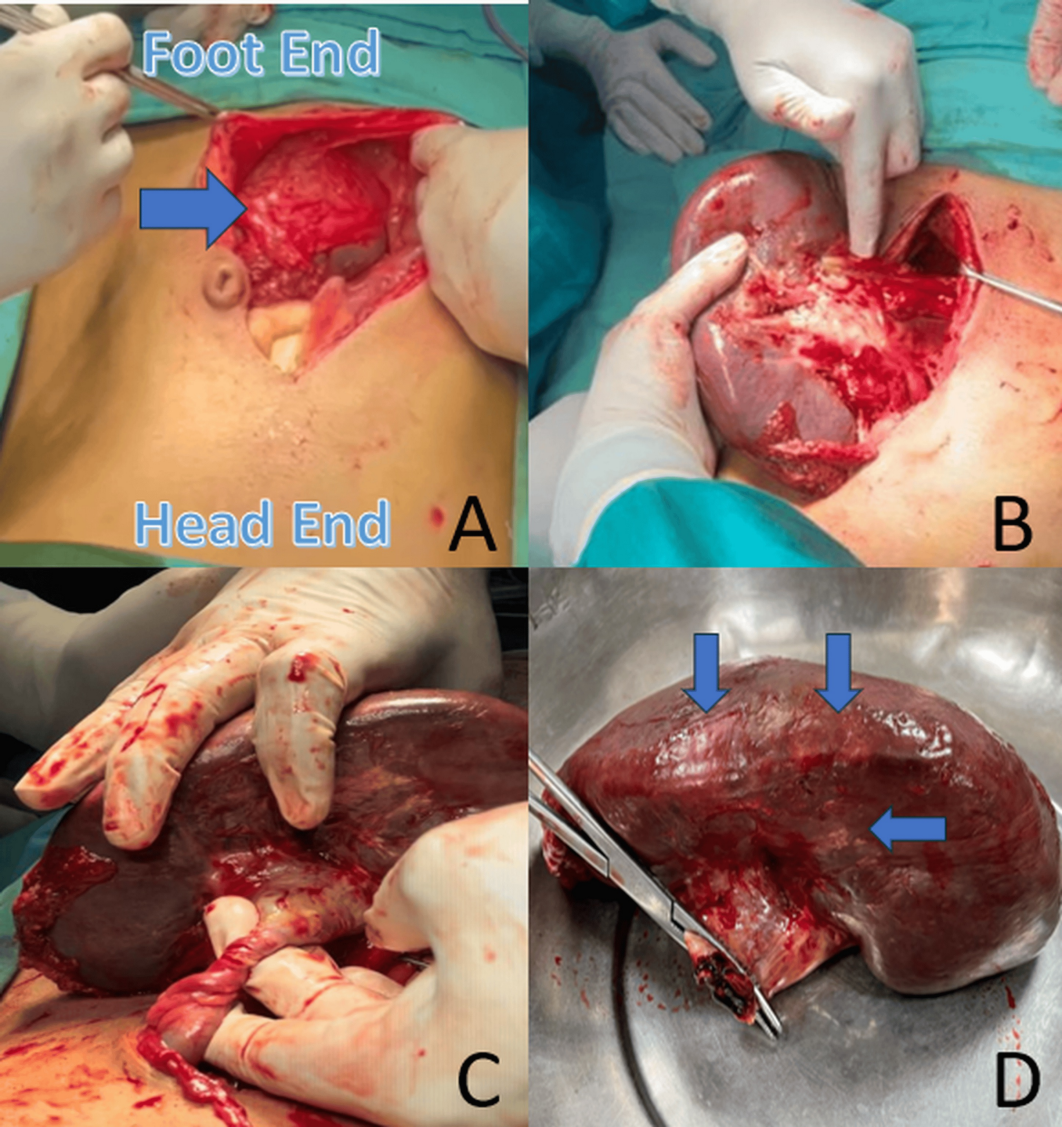
Intraoperative findings: (A) Shows the ectopically located spleen in the lower abdomen, confirming its displacement from the normal left upper quadrant. (B) Reveals a grossly enlarged and congested spleen with adherent bowel loops and signs of ischemia. (C) Highlights the torsion of an elongated splenic pedicle, which is the hallmark of a WS and leads to compromised blood flow. (D) Displays the excised spleen, showing congested, dusky areas of infarction, consistent with vascular compromise and infarction due to pedicle torsion WS: wandering spleen

Intraoperatively, the spleen was located in the right iliac fossa with dense adhesions to the sigmoid colon. These adhesions were meticulously dissected, and detorsion of the splenic pedicle was achieved. However, multiple infarcted areas were noted throughout the spleen, indicating irreversible ischemic damage. A total splenectomy was performed to prevent further complications. While splenectomy does not address the underlying portal hypertension in a patient with EHPVO, it is particularly beneficial in cases where hypersplenism is the dominant clinical problem (Figure 3).

The patient’s postoperative course was uneventful. He was discharged in stable condition with appropriate immunization against *Streptococcus pneumoniae*, Haemophilus influenzae type B, and *Neisseria meningitidis*. Long-term follow-up was advised. In view of the in situ biliary stent and underlying history of EHPVO, the patient was referred to the Department of Gastroenterology for further hepatobiliary evaluation. Magnetic resonance cholangiopancreatography (MRCP) and/or endoscopic retrograde cholangiopancreatography (ERCP) were recommended to assess for persistent infection, stent-related complications, or biliary obstruction. The patient was also counseled regarding the lifelong risk of overwhelming post-splenectomy infection (OPSI), the necessity of prompt medical attention for febrile illnesses, the importance of regular follow-up for hepatobiliary monitoring, and the potential need for biliary stent removal or exchange depending on gastroenterological evaluation.

## Discussion

The pathophysiology of WS involves the weakening or absence of the splenic suspensory ligaments namely the gastrosplenic, splenorenal, splenocolic, and phrenicocolic ligaments [1,2]. This anatomical defect allows the spleen to become mobile and susceptible to torsion of its vascular pedicle, which may compromise blood supply and lead to infarction or necrosis [7,11]. In children, this condition is usually congenital, whereas in adults it may be acquired due to trauma, connective tissue disorders, or hormonal effects. Notably, the hormone relaxin, which plays a role in softening ligaments during pregnancy, has been implicated in the acquired form of WS in women of reproductive age [5].

Another contributing factor is EHPVO, which leads to chronic portal hypertension and long-standing splenomegaly. This condition, commonly seen in non-cirrhotic portal hypertension, causes cavernous transformation of the portal vein and hypersplenism. The chronic increase in spleen size and weight exerts continuous mechanical stress on the ligaments, potentially resulting in secondary ligamentous laxity and subsequent WS [6]. Hence, EHPVO not only contributes to splenomegaly but may also act as a predisposing factor in the pathogenesis of WS.

CT remains the most effective diagnostic modality, capable of localizing the ectopic spleen and visualizing the “whirl sign” of pedicle torsion [11-13]. In the setting of EHPVO, CT can also demonstrate portal vein thrombosis, cavernous transformation, and collateral venous pathways. Although Doppler ultrasound can assess splenic perfusion and vessel patency, its diagnostic value is often limited by patient anatomy and acute abdominal conditions [11].

Treatment decisions are based on spleen viability. If the spleen is viable, detorsion followed by splenopexy preferably laparoscopic is the treatment of choice, as it preserves immune function and prevents recurrence [12]. In nonviable spleens, as in the present case, splenectomy is necessary to prevent complications such as rupture or abscess formation [13]. In EHPVO patients, splenectomy may alleviate hypersplenism, though careful evaluation is required due to the risk of aggravating portal hypertension and variceal bleeding.

Rarely, WS may be accompanied by volvulus of nearby organs, such as the small intestine, further complicating the clinical picture and necessitating urgent surgical intervention [10]. Clinicians should therefore maintain a high index of suspicion in patients with mobile abdominal masses, particularly women of reproductive age and individuals with longstanding splenomegaly. Early diagnosis and individualized surgical planning are essential for optimal outcomes [6,7,11-13].

## Conclusions

WS is a rare but potentially life-threatening condition resulting from abnormal splenic mobility due to ligamentous laxity or absence. This case highlights the importance of considering WS in the differential diagnosis of acute abdomen, particularly in patients with longstanding splenomegaly secondary to conditions like EHPVO. The association between EHPVO and WS underscores the mechanical impact of chronic splenomegaly on splenic ligament integrity. Prompt imaging, especially contrast-enhanced CT, is essential for diagnosis, enabling timely surgical intervention. Management must be tailored to spleen viability; in nonviable cases, as presented here, splenectomy remains the definitive treatment. Heightened clinical awareness and early intervention are critical to prevent complications such as infarction, rupture, or sepsis and to improve patient outcomes.
